# Alzheimer’s disease neuropathological change in younger individuals with IDH-mutant glioma

**DOI:** 10.1093/noajnl/vdaf057

**Published:** 2025-03-15

**Authors:** Lisa Greutter, Lisa Schnitzenlehner, Sigrid Klotz, Barbara Kiesel, Yelyzaveta Miller-Michlits, Jessica Makoli, Georg Widhalm, Bernhard Baumann, Adelheid Woehrer

**Affiliations:** University of Applied Sciences FH Campus Wien, Section Biomedical Sciences, Vienna, Austria; Comprehensive Center for Clinical Neurosciences and Mental Health – C³NMH, Division of Neuropathology and Neurochemistry, Medical University of Vienna, Vienna, Austria; Department of Neurology, Division of Neuropathology and Neurochemistry, Medical University of Vienna, Vienna, Austria; Comprehensive Center for Clinical Neurosciences and Mental Health – C³NMH, Division of Neuropathology and Neurochemistry, Medical University of Vienna, Vienna, Austria; Department of Neurology, Division of Neuropathology and Neurochemistry, Medical University of Vienna, Vienna, Austria; Comprehensive Center for Clinical Neurosciences and Mental Health – C³NMH, Division of Neuropathology and Neurochemistry, Medical University of Vienna, Vienna, Austria; Department of Neurology, Division of Neuropathology and Neurochemistry, Medical University of Vienna, Vienna, Austria; Comprehensive Center for Clinical Neurosciences and Mental Health – C³NMH, Division of Neuropathology and Neurochemistry, Medical University of Vienna, Vienna, Austria; Department of Neurosurgery, Medical University of Vienna, Vienna, Austria; Comprehensive Center for Clinical Neurosciences and Mental Health – C³NMH, Division of Neuropathology and Neurochemistry, Medical University of Vienna, Vienna, Austria; Department of Neurology, Division of Neuropathology and Neurochemistry, Medical University of Vienna, Vienna, Austria; Institute of Pathology, Neuropathology and Molecular Pathology, Medical University of Innsbruck, Innsbruck, Austria; Comprehensive Center for Clinical Neurosciences and Mental Health – C³NMH, Division of Neuropathology and Neurochemistry, Medical University of Vienna, Vienna, Austria; Department of Neurosurgery, Medical University of Vienna, Vienna, Austria; Comprehensive Center for Clinical Neurosciences and Mental Health – C³NMH, Division of Neuropathology and Neurochemistry, Medical University of Vienna, Vienna, Austria; Department of Neurosurgery, Medical University of Vienna, Vienna, Austria; Institute of Biomedical Physics, Medical University of Innsbruck, Innsbruck, Austria; Center for Medical Physics and Biomedical Engineering, Medical University of Vienna, Vienna, Austria; Institute of Pathology, Neuropathology and Molecular Pathology, Medical University of Innsbruck, Innsbruck, Austria; Comprehensive Center for Clinical Neurosciences and Mental Health – C³NMH, Division of Neuropathology and Neurochemistry, Medical University of Vienna, Vienna, Austria; Department of Neurology, Division of Neuropathology and Neurochemistry, Medical University of Vienna, Vienna, Austria

**Keywords:** Abeta, Aging, Alzheimer’s disease, IDH-mutant Glioma, pTau

## Abstract

**Background:**

With aging populations, the incidence of brain tumors and neurodegenerative diseases is rising. Recently, Alzheimer’s disease neuropathological change (ADNC) has been documented in the tumor-adjacent cortex of 50% of patients with glioblastoma, with isolated hyperphosphorylated tau (pTau) deposits already present in younger individuals. This study extends ADNC screening to younger patients with IDH-mutant glioma, focusing on pTau and amyloid beta (Abeta) deposits, microglial activation, and amyloid precursor protein (APP) expression in the context of cortical tumor cell infiltration.

**Material and Methods:**

We included 85 patients with IDH-mutant gliomas (37 astrocytomas, median age: 39; 48 oligodendrogliomas, median age: 50) classified as CNS-WHO grades 2–3. Tumor-adjacent cortex was immunohistochemically stained for b-A4, t-AT8, NeuN, APP, Ki67, and Iba1, and cells were quantified using Matlab and QuPath script. Longitudinal samples were available for 15 patients.

**Results:**

The median cell density in the tumor-adjacent cortex was significantly higher in glioma patients (astrocytoma: 1395/mm^2^, oligodendroglioma: 1492/mm^2^) compared to non-tumor cortex (1098/mm^2^, *P* < .0001). ADNC, including pTau (36%, *N* = 31/85) and Abeta (14%, *N* = 12/85), was observed in 38% (*N* = 32/85) of individuals. pTau and A beta positively correlated with age (Hazard ratio = 0.1, *P* = .02), tumor cell infiltration (Kendall’s tau = 0.100, *P* = 4.7*10^−4^), and diffuse axonal injury (*P* = .018). ADNC was commonly found in the temporal cortex (53%, *N* = 9/17).

**Conclusion:**

Our study reveals an unexpectedly high prevalence of ADNC, particularly isolated pTau deposits, in the tumor-adjacent cortex of younger individuals with IDH-mutant glioma. These findings suggest tumor-driven tau accumulation, prompting further research into potential long-term cognitive effects.

**Key Points**

- ADNC is present in tumor-adjacent cortex of 38% of patients with glioma, IDH mutant
- ADNC increases with patient age and varies according to the affected brain region
- Microglia response is highest in the presence of tumor cell infiltration plus pTau pathology and further modulated by patient age

**Importance of the Study**

Our study conducted a thorough examination of ADNC in the tumor-adjacent cortex of patients with IDH-mutant glioma. We found ADNC in about one third of these patients, with variations according to age and brain region. Our results particularly highlight isolated pTau deposition already at young age, suggesting important avenues for research into the interplay between tumor biology and secondary neurodegeneration. Given the prolonged survival of affected individuals, our findings underscore the potential relevance of detailed cognitive assessments and eventually pTau-directed therapies to mitigate cognitive decline.

Isocitrate dehydrogenase mutant gliomas (IDH-mutant glioma) are malignant diseases of the brain that affect younger individuals with peak age between 20 to 40 years.^1^ They comprise astrocytoma and oligodendroglioma tumor types, which share the IDH mutation but differ by ATRX inactivation and 1p 19q deletion status. Grading is based on combined histopathological and molecular features from 2 (lowest) to 4 (highest grade).^1,2^

Alzheimer’s disease (AD) is the most prevalent neurodegenerative disorder, which sporadically affects individuals over 65 years of age.^3^ AD is characterized by disrupted proteostasis leading to inadequate lysosomal degradation and the accumulation of amyloid beta (Abeta) and hyperphosphorylated tau (pTau) proteins. These protein deposits result in neuronal loss and a gradual decline in cognitive function ultimately progressing to dementia.^4,5^ The pathophysiology of AD is intricately linked with cellular changes in the cortical microenvironment including reactive proliferated and exhausted glia and microglia, which play a crucial role in disease progression.^6,7^

Beyond inflammation, preclinical studies proposed direct biological mechanisms that link glioma with ADNC.^8–10^ Precisely, pTau was reported in IDH-mutated gliomas, where high expression was found to stabilize microtubules and inhibit mesenchymal transformation and angiogenesis, thereby reducing tumor aggressiveness and improving chemotherapy sensitivity.^8^ In a recent study, we observed AD neuropathological changes (ADNC) in tumor-adjacent cortex of about 50% of patients with glioblastoma.^11^ While the co-occurrence did not exceed age-matched controls in the elderly, an excess risk for isolated pTau deposits was suggested in younger individuals below age 40. To investigate this further, we here focused on a younger patient population, i.e. adult-type IDH-mutant glioma. In our approach, we utilize a representative in vivo and postmortem cohort of patients with IDH-mutant glioma to systematically screen tumor-adjacent cortex for ADNC.

## Materials and Methods

### Patients and Tissues

**Figure 1.**
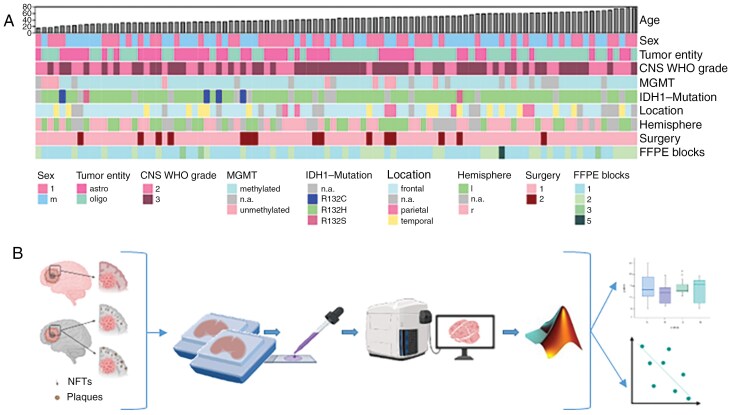
Cohort demographics and workflow. (A) Heatmap detailing clinical factors sorted by ascending patient age. (B) Schematic illustration of glioblastoma-adjacent cortex displaying ADNC (pTau positive neurofibrillary tangles (NFTs) and/or Abeta plaques) and workflow of tissue embedding, histological processing, image digitization, segmentation, automated quantification, statistical analysis and data visualization. Created with BioRender.com.

We included 140 formalin-fixed and paraffin-embedded (FFPE) tissues of 85 patients with low-grade glioma, IDH-mutant, CNS-WHO grade 2–3, diagnosed between 2002 and 2023 (37 astrocytomas IDH mutant, CNS-WHO grade 2–3, median age: 39; 48 oligodendrogliomas, IDH mutant and 1p/19q co-deleted, CNS-WHO grades 2–3, median age: 50). Additionally, 5 brain autopsies from patients with astrocytoma or oligodendroglioma were examined. Patient age ranged from 15 to 77 years with a median age of 45.2 years (Figure 1A). The female-to-male ratio was 1.07. Inclusion criteria comprised the presence of tumor and adjacent cortex upon histology. Tissues were retrieved from the neurobiobank of the Medical University of Vienna and patient demographics were abstracted from clinical records (summarized in Figure 1A). Tumor location was most commonly frontal (*N* = 53/85, 62%), followed by temporal (*N* = 14/85, 16%) and parietal (*N* = 5/85, 6%) with a preference for the right hemisphere (ratio 37:30). For the remaining 13 patients, information on location was not available and for 18 patients, the hemispheric side was missing. Longitudinal samples from first and second surgeries were available for 15 patients. Out of 85 samples, 83 exhibited IDH1 mutations, while IDH2 mutations were absent, and for the remaining two samples, the IDH status was not available. The types of IDH1 mutations varied, including R132H, R132S, and R132C. This study was conducted in accordance with the ethical standards outlined in the Declaration of Helsinki. Ethical approval was obtained from the Institutional Review Board at Medical University of Vienna (EK 1429/2022, EK 1375/2018), and written informed consent was waived due to the retrospective character of the study.

### Tissue Processing

The entire workflow is shown in Figure 1B. FFPE samples were cut at a thickness of 2 µm followed by immunohistochemistry using the following antibodies: anti-NeuN (EMD Millipore, USA, clone MAB377, 1:2000, mouse, heat-induced epitope retrieval (HIER) pH6), anti-b-A4 (DAKO, Denmark, clone 6F/3D, 1:100, mouse, 80 % formic acid (FA) 1 h), anti-tau-AT8 (Invitrogen, Belgium, 1: 200, clone AT8 pS202/pT205, mouse, no pretreatment), anti-APP (EMD Millipore, USA, clone 22C11, 1:8000, mouse, HIER pH6), anti-tau-RD3 (EMD Millipore, USA, clone 8E6/C11, 1:16000, mouse, HIER pH6 plus FA 100% 1 min), anti-tau-RD4 (EMD Millipore, USA, clone 1E1/A6, 1:800, mouse, HIER pH6 plus FA 100% 1 min), anti-Ki67 (DAKO Mib-1, Denmark, 1:200, mouse, HIER pH9) and anti-Iba1 (Wako, Japan, 1:1000, rabbit, HIER pH6). Staining was performed using the Leica DAKO Autostainer Link 48 with DAKO Envision System Kit (DAKO, Glostrup, Denmark) for mice-anti-human and rabbit-anti-human primary antibodies, using diaminobenzidine as chromogen. The incubation time of the primary antibodies was 30 min for anti-NeuN, anti-tau-AT8, anti-tau-RD3, anti-tau-RD4, anti-Ki67, and anti-APP, 60 min for anti-b-A4, and 2 h for anti-Iba1 at room temperature. Slides were mounted and digitized using a Hamamatsu Nanozoomer 2.0 HT slide scanner at a magnification of 40×. Double immunofluorescence stainings with APP and IDH1 as well as APP and NeuN were performed to evaluate the APP expression in neurons and tumor cells. OPAL 690 and OPAL 620 were used as fluorescence markers, APP and NeuN were used as described above. IDH1 (Dianova, Germany, Clone H09, ready to use, mouse, HIER pH6, 30min at room temperature) and NeuN were labeled with OPAL 690, APP with OPAL 620. The slides were mounted and digitized using an Akoya Vectra Polaris platform.

Five postmortem brains of individuals with IDH-mutant glioma were analyzed (median age 53 years). In each case, ground truth AD stages were assessed based on the following regions: hippocampus, middle and superior temporal gyrus, middle frontal gyrus, parietal med/inf gyrus, occipito-medial cortex, basal ganglia, thalamus, pons, medulla oblongata, and cerebellum. In addition, tumor-adjacent cortex was randomly sampled and included the following regions: temporal cortex (*N* = 2), frontal cortex (*N* = 1), insular region (*N* = 1), and brainstem (*N* = 1). All blocks were cut and stained for anti-b-A4 and anti-t-AT8. The median Braak & Braak stage was I (range 0–III), the median Thal phase was 0 (range 0–1), and the median CERAD score was 0 (range negative-0, thus including cases with diffuse plaques only). Notably, two patients exhibited isolated threads in the entorhinal cortex and one patient showed no ADNC.

As controls, we utilized 3 specimens obtained during epilepsy surgery to assess diffuse axonal injury (DAI) in the absence of tumor. Those samples were cut and stained for APP.

### Semiquantitative Neuropathological Scoring

In the digital slides, both the cortex and tumor tissue were carefully delineated through manual segmentation using the NDPI-viewer. Semiquantitative scoring was done by S.K. and L.G. The extent of tumor cell infiltration into the cortex was scored into three categories based on the proportion of hematoxylin-stained cells observed on the anti-NeuN-stained slide. Low: the cortical area mostly consisted of neurons (Supplementary Figure 1A1). Medium: Approximately 50% neurons and 50% non-neuronal cells present (Supplementary Figure 1A2). High: more than 50% non-neuronal cells (Supplementary Figure 1A3). Abeta deposits were categorized based on CERAD criteria: negative: absence of deposits. Stage 0: presence of diffuse plaques only (Supplementary Figure 1B1). Stage A: few neuritic plaques (3–5 per high-power field; Supplementary Figure 1B2). Stage B: moderate neuritic plaques (5–10 per high-power field; Supplementary Figure 1B3). Moreover, the presence of amyloid deposits in vessel walls and in capillaries (cerebral amyloid angiopathy, CAA) was evaluated and classified as either present or absent. The accumulation of pTau in the cortex was scored according to the amount of pTau positive deposits into 4 categories (0 = negative; 1 = threads; 2 = single NFTs and threads (1 per high-power field); and 3 = NFTs and threads (3 and more per high-power field), Supplementary Figure 1C). We refrained from using Braak stages since the pTau load was sparse for the majority of cases. All ADNC-related parameters were independently scored by two experts (S.K. and L.G.). Cohen’s Kappa was calculated to assess inter-rater agreement (Supplementary Table 1). The presence or absence of secondary Scherer structures was evaluated for each case.^12^ The expression of amyloid precursor protein (APP) by tumor cells and neurons, as well as the extent of DAI were semiquantitatively assessed. Tumor cell and neuronal expression of APP was scored based on the percentage of cells showing expression: sparse: 1% to 5% (Supplementary Figure 1D1); moderate: 10% to 30% (Supplementary Figure 1D2 and E1); frequent: more than 50% (Supplementary Figure 1D2, E2, and 1E3). DAI was classified into four categories: none: no axonal spheroids were observed; sparse: 1 to 10 axonal spheroids were present per high-power field (Supplementary Figure 1F1); moderate: 10 to 30 axonal spheroids were observed (Supplementary Figure 1F2); frequent: more than 30 axonal spheroids were seen (Supplementary Figure 1F3). Representative images are showcased in Supplementary Figure 1.

### Automated Image Analysis

NeuN stainings were segmented into cortical and tumor tissue using the NDPI.viewer at the level of whole slide scans. Using the NeuN-based cortical segmentations, the area of cortex and fractions of neuronal and non-neuronal cells in these regions were quantified. Images of NeuN, Abeta, pTau, and Iba1 stainings were registered upon each other and pixel-based analysis for cell counting in Matlab was done as previously described.^11^ Ki67 stainings were loaded into QuPath, and a quantification of Ki67-positive cells was established using the positive cell detection routine. Cell counts and area measurements were extracted for further analysis in R.^13^

### Group-Level Statistical Analysis and Data Visualization

In the data analysis, R-Studio was utilized for statistical and graphical analyses. ANOVA with pairwise *t*-tests using the Bonferroni method was applied to compare the medians of different groups. Shapiro–Wilk tests were employed to assess normal distribution. If the *P*-value was below .05, data was not normally distributed. To predict the *P*-values of correlations involving normally distributed variables, Pearson’s correlation was employed, while Kendall’s tau was utilized for variables that were not normally distributed. For the analysis of contingency tables, Chi^2^-test was utilized. To examine the associations between Abeta and/or pTau with age, location, and the other marker, logistic regression analysis was performed. Associations between microglial counts and ADNC were assessed using odds ratios. To explore the impact on overall survival, Cox regression analysis was conducted, and the results were visualized using Kaplan–Meier plots. Cohen’s Kappa was incorporated to analyze inter-rater variability. To maintain robustness, the alpha value was set at the conventional threshold of 0.05.

## Results

### Cortical Tumor-Infiltration is Associated With Neuronal Loss

**Figure 2.**
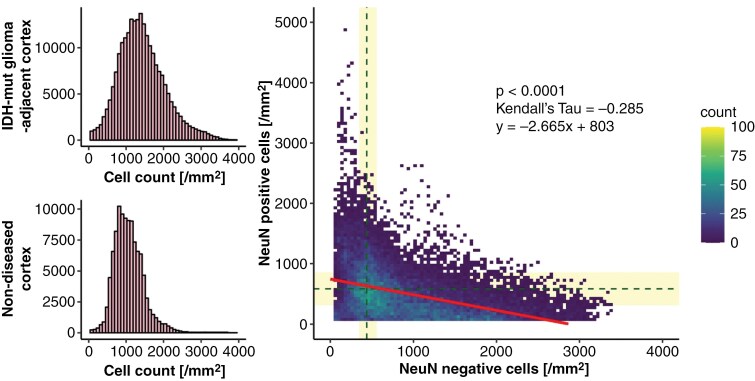
Cortical invasion by glioma IDH mutant is associated with neuronal loss. (A) Cell counts per mm^2^ in cortices adjacent to IDH-mut gliomas (upper panel, median = 1444 cells/mm^2^, *N* = 85) and in the healthy cortex of age-matched postmortem brains (lower panel, median = 1098 cells/mm^2^, *N* = 5 control brains, *P*-value = 2*10^−16^). (B) The scatterplot illustrates the distribution of NeuN-positive neurons (y-axis) and NeuN-negative non-neuronal cells in the cortex. Median values from 3 postmortem control brains are indicated by dotted lines, with bars representing confidence intervals (Kendall’s tau = −0.285, *P*-value = 1.2*10^−15^, *N* = 85).

In the entire cohort, cortical regions of high, medium, and low tumor-infiltration were equally distributed upon semiquantitative scoring with considerable within-patient variability (Supplementary Figure 2A). A median of 110.9 mm^2^ (range 0.3 to 711.5 mm^2^) of cortical area was present per patient. Within the tumor-adjacent cortex, a median of 1,444 cells per mm^2^ (range 556 to 10,519 cells) was quantified (Figure 2A). Compared to non-tumor-infiltrated cortex of three postmortem brains with mild edema only, median cell counts were significantly higher (Figure 2B, 1,098 versus 1,444 cells/mm^2^, *P*-value = 2*10^−16^). No significant differences in cell counts were observed between different cerebral lobes or hemispheres, or between females and males. Overall, the fraction of neurons decreased with increasing content of non-neuronal cells (Kendall’s tau = −0.285, *P*-value = 1.2*10^−15^, Figure 2C). This phenomenon was observed in both astrocytoma and oligodendroglioma (Supplementary Figure 2B).

### ADNC in Glioma-Infiltrated Cortex

**Figure 3.**
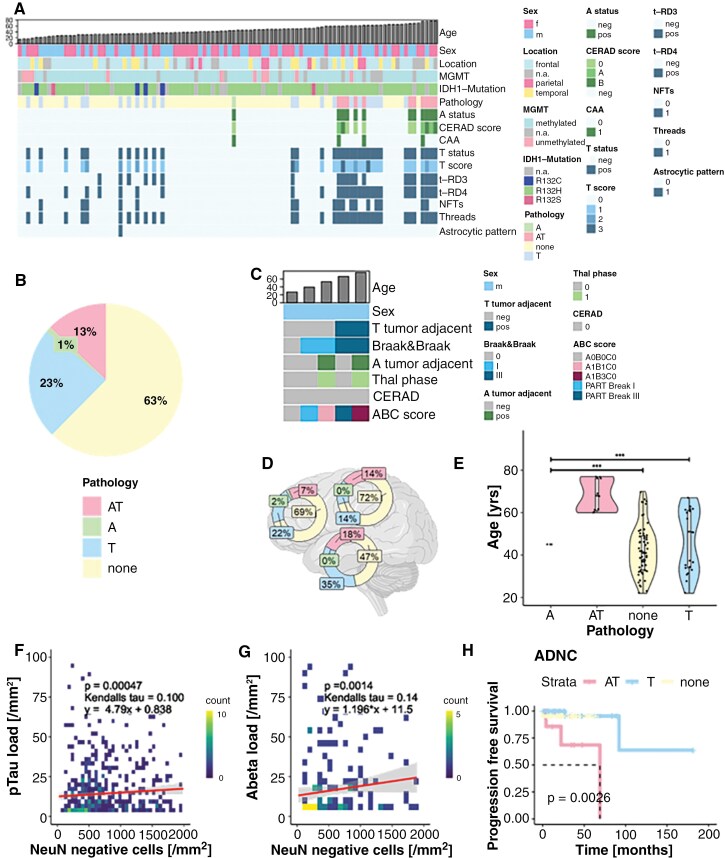
Neuropathological mapping of ADNC. (A) Heatmap of Abeta (A) and pTau (T) deposits per patient sorted by age (*N* = 85). (B) Distribution of ADNC (*N* = 85). (C) Heatmap of a postmortem IDH-mut glioma cohort (*N* = 5) with ADNC ratings for random tumor-adjacent cortex in comparison to ground truth Braak & Braak stages, Thal phases and CERAD scores. (D) Topographic differences among ADNC patterns (*N* = 71), the color scheme fits to B. (E) Boxplot of the age range per protein deposits (*P*-value_AT-T_ = 2.3*10^−5^, *P*-value_AT-none_ = 2.7*10^−8^, *P*-value_T-none_ = .66, *N* = 85). (F) 2D-histogram of pTau load against tumor cell density in the cortex (*N* = 31, *P*-value = 4.7*10^−4^, Kendalls tau = 0.1). (G) 2D-histogram of Abeta load against tumor cell density in the cortex (*N* = 12, *P*-value = .0014, Kendalls tau = 0.14). (H) ***Kaplan Meier analysis stratified into ADNC*** (*N* = 62, *P*-value = .0026).

**Figure 4.**
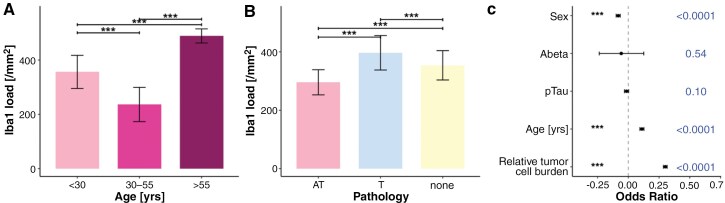
Microglia load according to age and ADNC protein deposits. (A) Iba 1 counts according to age groups (*N* = 85, *P*-value_<30vs30-55_ = 2.1*10^−16^; *P*-value_<30vs>55_ = 1.7*10^−9^; *P*-value_30-55vs>55_ = 6.1*10^−12^). (B) Iba1 counts according to ADNC (*N* = 85, *P*-value_ATvsT_ = 7.6*10^−11^; *P*-value_ATvsnone_ = 4.5*10^−10^; *P*-value_Tvsnone_ = 8.4*10^−7^). (C) Forrest plot of Pearson correlations for the effect of each variable on Iba1 count per mm^2^. Female sex was associated with significantly higher microglial counts (*N* = 85).

A total of 40% (*N* = 34/85) of the patients showed any ADNC in tumor-infiltrated cortex with substantial between- and within-subject variability (Supplementary Figure 3 and Figure 3A). More specifically, 26% (*N* = 22/85) had isolated pTau deposits, followed by 13% (*N* = 11/85) with combined Abeta and pTau deposits including 4 with additional vascular Abeta deposits (2/4 in cappilaries, Allen type 3, Thal type 1), and one case with isolated Abeta plaques (1%). 60% (51/85) had no additional ADNC (Figure 3B). Among samples with isolated pTau deposits, 13 showed enough pTau positivity to analyze 3 and 4 repeat tau isoforms, which yielded a mixture of 3 repeat and 4 repeat isoforms consistent with AD in 62% (*N* = 8/13). In the remaining 38% (*N* = 5/13) only the 4 repeat tau isoform was found potentially indicating the presence of further, more specific tauopathies.^14^ Astrocytic tau in the form of fuzzy astrocytes was reported in three of 22 (14%) cases. Isolated pTau and combined Abeta and pTau deposits were most common in the temporal lobe (Figure 3D) following the endogenous early involvement in AD progression. In an attempt to better characterize the sampling error due to random picks of tumor-adjacent cortices, we leveraged a postmortem cohort of patients with glioma, IDH mutant with ground-truth ADNC. Randomly selected tumor-adjacent cortical samples demonstrated concordant Abeta results in 5 of 5 patients, while pTau was less consistently sampled (detected in 3 of 5 patients) with early Braak stages I–II being undetected in the cortical samples from non-temporal i.e. frontal lobes (Figure 3C). The glioma type did not significantly impact ADNC, even though patients with oligodendroglioma were more often affected (40% as compared with 23%, Supplementary Fig 4A and B, *P*-value = .25; Chi^2^=3.89). Likewise, the CNS-WHO grade did not exert a significant influence on the presence of AD-related proteins; however, a trend toward lower grade being associated with increased ADNC was observed (Supplementary Fig 4A and B, *P*-value = .21; Chi^2^ = 6.50). The probability of ADNC increased with age. The group with combined Abeta and pTau (AT) ADNC was significantly older than patients with pTau only (T) or without ADNC (*P*-value_AT-T_ = 2.3*10^−5^, *P*-value_AT-none_ = 2.7*10^−8^, *P*-value_T-none_ = .66, Figure 3E). Higher tumor-infiltration into the cortical regions was associated with elevated pTau values (Figure 3F, *N* = 31, *P*-value = 4.7*10^−4^, Kendall’s tau = .1) and Abeta load (Figure 3G, *N* = 12, *P*-value = .0014, Kendall’s tau = 0.14). For a subset of 15 individuals, matched recurrent tumors were available for repeated AD screening. The median patient age at diagnosis was 36 years and the median time between the first and second surgery was 5.3 years. Two of these cases showed isolated pTau deposits. All 15 cases displayed stable pathology (Supplementary Figure 4C). Logistic regression revealed that CERAD scores were significantly associated with age and pTau scores (*P*-value_age_ = 2.51*10^−5^, *P*-value_pTauScore_ = .0012), and pTau scores with temporal location and CERAD score (*P*-value_temporal_ = .003; *P*-value_CERAD_ = .001; Supplementary Figure 4D and E). To explore a potential excess risk of ADNC in patients with IDH-mutant glioma, a direct comparison with eleven major population-based studies was performed (PMIDs provided in Supplementary Figure 4F and G), which suggested that the observed Abeta frequencies were within the expected age range. In contrast, pTau deposits exceeded those of age-matched controls in younger individuals (note: only one population-scale study focused on younger individuals, which utilized silver staining to differentiate pre-tangles from NFTs, as referenced in Supplementary Figure 4F and G). Progression-free survival (PFS) of the patients was not influenced by the presence of additional Abeta or pTau pathology. Upon univariate survival analysis ADNC and pTau scores were significantly associated with worse PFS, while CERAD scores had no significant impact (Figure 4H, Supplementary Figure 4 H and I; *P*-values: ADNC = .0026, pTau = .0089, CERAD = .53).

When adjusting for age, sex, and tumor type, presence of ADNC (*P* = .0026) higher pTau load (pTau Score = 2, *P* = .0089), and CERAD scores (A and B, *P* = .53) did not contribute independent information on survival (Supplementary Figure 4J; *P*-values: pTau = .71, CERAD = .07, age = .06, male sex = .94, oligodendroglial tumors = .49).

In a second step secondary structures of Scherer were analyzed.^12^ Out of the 85 patients, all showed perineuronal satellitosis (Scherer A). Perivascular satellitosis (Scherer B) was observed in 68 out of 85 cases (80%). Subpial spread (Scherer C) of glioma cells was noted in 37 individuals (44%), while invasion of glioma cells along white matter tracts (Scherer D) was reported in 47 patients (55%). Respective histological pictures are shown in Supplementary Figure 5A–D. A correlation analysis was conducted to explore the relationship between each Scherer structure and ADNC. However, none of these structures were found to significantly impact ADNC (Scherer B: Chi^2^ = 1.799, *P*-value = .615; Scherer C: Chi^2^ = 0.844, *P*-value = .838; Scherer D: Chi^2^ = 5.479, *P*-value = .139, Supplementary Figure 5A–H).

The impact of IDH1-mutations on ADNC was also analyzed. The results indicate that IDH1 mutations do not influence the presence of ADNC (Chi-squared = 8.7704, *P*-value = .4587, Supplementary Figure 5I). In a last step the impact of tumor proliferation on ADNC was analyzed using Ki67-IHC stainings. No statistically significant differences were identified between the four ADNC-groups (*P*-value = .17, Supplementary Figure 5J). In a subsequent analysis, the impact of Ki67 proliferation index on the pTau score and CERAD score was examined. A higher number of Ki67-positive cells was not associated with higher pTau or CERAD scores (pTau: *P*-value = .11; CERAD: *P*-value = .12, Supplementary Figure 5K and L). It should be noted that the number of cases with higher pTau and CERAD scores was limited, which may have affected the statistical power.

### Microglial Activation in Response to Cortical Tumor-Infiltration Plus ADNC

Iba1 load correlated positively with age (*P*-value < .0001, Figure 4A and C). The highest degree of microglial activation was found in individuals with pTau deposits (all *P*-values < .0001, Figure 4B). Microglial load correlated with extent of tumor-infiltration (odd´s ratio (OR) = 0.301; 95%CI = 0.285 to 0.316), patient age (OR = 0.111, 95%CI = 0.0940 to 0.127), and sex (OR = −0.014; 95%CI = −0.031 to 0.0028), whereas pTau and Abeta had non-significant effects (OR_Abeta_ = −0.057, 95%CI = −0.236 to 0.125; OR_pTau_ = −0.0140, 95%CI = −0.031 to 0.0028).

### A Fraction of Tumors Express APP But Many Show DAI

**Figure 5.**
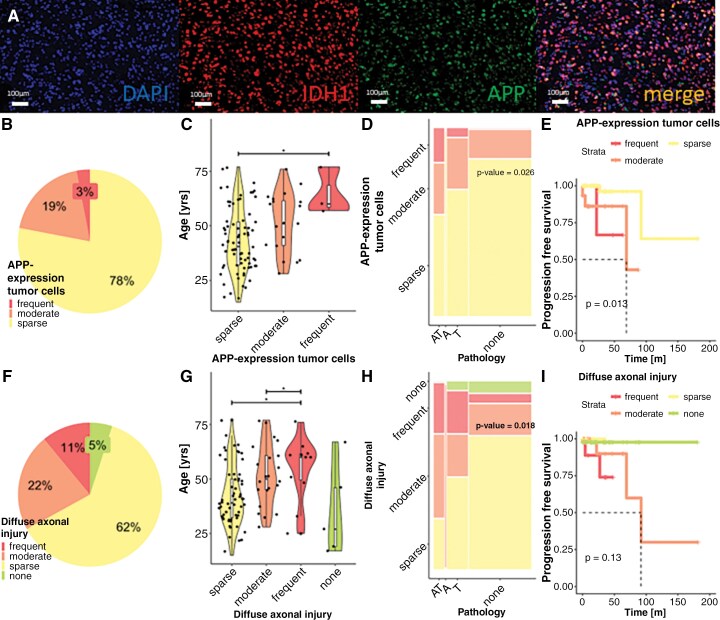
Amyloid precursor protein (APP) expression and diffuse axonal injury (DAI). (A) APP expression by tumor cells (scale bar = 25µm). Double-staining of APP and IDH1, all scale bars = 100µm. (B) Prevalence of APP expression in tumor cells. (C) Effect of age on APP expression in tumor cells (*N* = 85, *P*-value_sparse-frequent_ = .036; *P*-value_moderate-frequent_ = .46; *P*-value_sparse-moderate_ = .055). (D) Effect of APP expression by tumor cells on ADNC (*N* = 85, *P*-value = .026). (E) Kaplan–Meier analysis for APP expression in tumor cells (*N* = 62, *P*-value = .69). (F) Prevalence of DAI (*N* = 85). (G) Effect of age on DAI (*N* = 85, *P*-value_sparse-moderate_ = .048; *P*-value_sparse-frequent_ = .047; *P*-value_sparse-none_ = 1; *P*-value_moderate-frequent_ = 1; *P*-value_moderate-none_ = .14; *P*-value_frequent-none_ = .078). (H) Effect of DAI on ADNC (*N* = 85, *P*-value = .013). (I) Kaplan–Meier analysis for DAI (*N* = 62, *P*-value = .13).

APP, the precursor molecule for Abeta, was expressed in 22% of all tumors, with 3% showing expression in over 50% of tumor cells (Figure 5A and B). APP expression was related to patient age (Figure 5C, *N* = 85), with a median age of 60 years for the frequent expression group, 51 years for the moderate group, and 40.5 years for the sparse group. Significant differences were observed between the sparse and frequent groups (*P*-value = .036), while the differences between the other groups were not statistically significant. Additionally, APP expression correlated with the presence of ADNC (Figure 5D, *P*-value = .026), while it had no impact on the PFS (Figure 5E, *P*-value = .69). APP expression in tumor cells was not influenced by CNS-WHO grade or glioma type (Supplementary Figure 6A; *P*-value_CNSWHOgrade_ = .91; *P*-value_Tumor type_ =.10). In all cases, cortical neurons consistently exhibited APP expression.

The accumulation of APP in axons is a well-established marker for DAI, which is detectable from 2–3 hours up to 9 months after axonal damage.^15^ Topographically, DAI was observed in and around necrotic tumor regions. It was frequent in 11% (*N* = 9/85) of the cases, moderate in 22% (*N* = 19/85), and sparse in 62% (*N* = 53/85, Figure 5E). DAI was age-dependent (Figure 5F), with significant differences observed between the sparse and moderate groups (*P*-value = .048) and between the sparse and frequent groups (*P*-value = .047). Additionally, DAI was associated with the presence of ADNC (Figure 5G, *P*-value = .018). Its presence did not affect PFS (*P*-value = .39, Figure 5H), and it was not correlated with CNS-WHO grade or tumor type (Supplementary Figure 6B; *P*-value_WHOgrade_ = .29; *P*-value_Tumor_ = .37). A trend toward more severe DAI was observed in higher tumor grades, particularly in oligodendroglioma.

### Comparison With Glioblastoma

**Figure 6.**
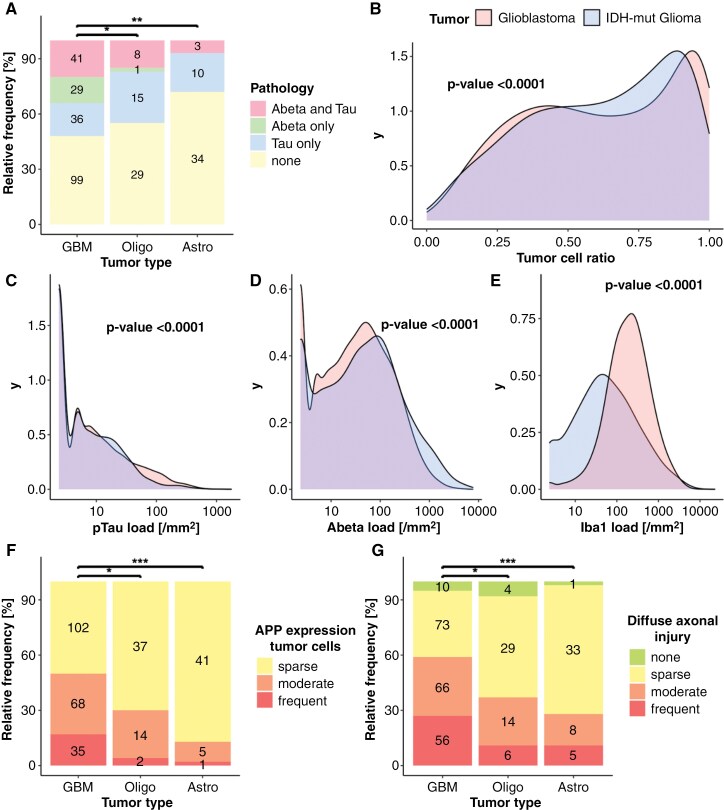
Differences between IDH-mutant glioma and IDH-wt glioblastoma. (A) Differences in appearance frequencies of ADNC in glioblastoma, oligodendroglioma and astrocytoma (*N* = 290, *P*-value_astrocytoma-glioblastoma_ = 1.7*10^−5^, *P*-value_oligodendroglioma-glioblastoma_ = 1.1*10^−5^, *P*-value_astrocytoma-oligodendroglioma_ = 1). (B) Density curve for differences in cortical tumor-infiltration between glioblastoma and IDH-mutant glioma (*P*-value = 2.2*10^−16^). (C) Density curve of pTau distribution (*P*-value = 2.2*10^−16^). The color scheme refers to B. (D) Density of Abeta load distribution in IDH-mut glioma and glioblastoma (*P*-value = 2.2*10^−16^). (E) Density of Iba1 positive microglia (*P*-value = 2.2*10^−16^). (F) Differences in appearance frequencies of amyloid precursor protein expression in tumor cells in glioblastoma, oligodendroglioma and astrocytoma (*N* = 290, *P*-value_astrocytoma-glioblastoma_ = 1.6*10^−5^, *P*-value_oligodendroglioma-glioblastoma_ = .011, *P*-value_astrocytoma-oligodendroglioma_ = .33). (G) Differences in appearance frequencies of diffuse axonal injury in glioblastoma, oligodendroglioma and astrocytoma (*N* = 290, *P*-value_astrocytoma-glioblastoma_ = 2.8*10^−4^, *P*-value_oligodendroglioma-glioblastoma_ = .025, *P*-value _astrocytoma-oligodendroglioma_ = .11).

Compared to recent work in glioblastoma,^11^ we observed significant differences in the frequency of ADNC (*P*-value_astrocytoma-glioblastoma_ = 1.7*10^−5^, *P*-value_oligodendroglioma-glioblastoma_ = 1.1*10^−5^, *P*-value _astrocytoma-oligodendroglioma_ = 1, Figure 6A) with fewer Abeta deposits and decreased pTau load in lower grade glioma. However, pTau deposits were found in much younger individuals as compared with glioblastoma (age ranges are plotted in Supplementary Figure 7A). Both, cortical tumor cell infiltration (*P*-value = 2.2*10^−16^, Figure 6B) and microglial response were higher in glioblastoma (343 Iba1 positive cells/mm^2^ as compared to 143 cells/mm^2^, *P*-value = 2.2*10^−16^, Figure 6D). Likewise, APP expression by tumor cells was higher in glioblastoma (*P*-value_astrocytoma-glioblastoma_ = 1.6*10^−5^, *P*-value_oligodendroglioma-glioblastoma_ = .011, *P*-value _astrocytoma-oligodendroglioma_ = .33, Figure 6E) and the same was true for the presence of DAI highlighting the aggressive growth of glioblastoma (*P*-value_astrocytoma-glioblastoma_ = 2.8*10^−4^, *P*-value_oligodendroglioma-glioblastoma_ = .025, *P*-value _astrocytoma-oligodendroglioma_ = .11, Figure 6F). The prevalence of ADNC was also compared in age-matched groups of both tumors. Abeta was present in 11% of patients with glioblastoma IDH-wt aged 40 to 50 years (*N* = 1/9), and in 18.5% of those aged 50 to 60 years (*N* = 5/27), while it was almost absent in age-matched cases of IDH-mutant glioma. On the other hand, pTau was similar or tended to be more common in IDH-mutant glioma across all age groups (Supplementary Figure 7B).

## Discussion

In this study, we utilized a large cohort of patients with IDH-mutant glioma to systematically screen tumor-adjacent cortex for the presence of ADNC. The most striking finding was the high prevalence of ADNC, particularly isolated pTau deposits, in up to 40% of the patients, despite their relatively younger age. This frequency of pTau deposits seemed to exceed age-matched controls in younger patients.^16–26^ Given that pTau deposits per area [mm^2^] also increased with the extent of cortical tumor cell infiltration, a secondary, tumor-induced tau pathology seems likely, potentially similar to chronic traumatic encephalopathies (CTE), where tau pathology is induced by small repetitive traumas.^27^ Tau pathology was not influenced by IDH mutation, tumor-proliferation score, or secondary Scherer structures. Similarly, in our study, pTau deposition was prominent in areas neighboring the tumor. pTau deposits, which correlate with cognitive function,^28,29^ were predominantly found in the temporal and frontal cortices, thus corresponding to early to intermediate Braak stages in AD (not fully applicable here, since often isolated pTau without Abeta). Although the overall pathological burden was relatively low and cognitive function was not systematically evaluated in our patient cohort, our findings raise the concern of long-term risk for cognitive decline due to pTau seeding and progression later in life. However, given the descriptive nature of this study, the subtlety of the pTau deposits, and the lack of further imaging or liquid-based biomarkers, these results should be interpreted cautiously.

Another driver of pTau spread in CTE or Alzheimer’s disease is microglial activation,^30^ which is also prominent in the tumor setting. Not surprisingly though, patients with IDH-mutant glioma plus pTau deposits had the highest microglial load. However, multivariate analysis suggested that the impact of the tumor cells on microglial activation was higher as compared to pTau deposits.

In CTE, tau pathology was linked to episodes of DAI, which is characterized by APP accumulation in axonal spheroids, which form within 2–3 hours after acute axonal disconnection.^31^ This axonal injury can disrupt axonal transport, leading to the release of tau from microtubules.^30^ We observed distinct DAI patterns, particularly in the tumor and around areas of tumor necrosis, with more severe DAI notably present in grade 3 oligodendrogliomas, where necrosis was present.^2^ Notably, their exclusive presence in tumor specimens as opposed to epilepsy surgical samples, suggests a tumor-induced phenomenon rather than due to surgical dissection. In this series, both APP expression by tumor cells and DAI were correlated with the presence and extent of ADNC, potentially indicating causal involvement.

In comparison to glioblastoma IDH-wildtype, patients with IDH-mutant gliomas demonstrated fewer ADNC deposits, fewer microglial load, and less axonal injury,^11^ which may well be attributed to the less aggressive growth patterns with fewer necroses as well as a younger age of the patients.^1^

Treatment-induced accelerated brain aging is an emerging area of concern with significant implications for patients undergoing chemotherapy and radiotherapy.^32,33^ Given that patients with IDH-mutant glioma often undergo intensive multimodal treatment and may already present with ADNC at a younger age,^34^ we anticipated a longitudinal increase in ADNC over time, which was not evident in our preliminary set of 15 cases. However, due to the limited availability of longitudinal tissue samples containing tumor-adjacent cortex, our study was underpowered to effectively assess longitudinal changes of ADNC.

Our study has several limitations. First, the availability of cortical regions for screening was constrained by tumor location, which hindered a comprehensive staging of AD. Additionally, we lacked cognitive assessments, and AD biomarkers to evaluate the impact of ADNC on patient cognition. Neither demographic data such as diabetic status, weight, and blood pressure nor MR-images were accessible. Second, our custom digital image analysis pipeline depended on multiple serial sections per tissue block. Despite meticulous registration of heat maps, slight misalignments between corresponding regions may have influenced our correlative analysis. To address notable staining variability between slides and samples, we employed a labor-intensive manual color-marking technique. Future studies would benefit from more advanced methodologies, such as multiplex stainings and enhanced segmentation tools to fine-map delicate structures like individual pTau threads.

In conclusion, our study reveals a high prevalence of ADNC, particularly pTau deposits, in the tumor-adjacent cortex of patients with IDH-mutant glioma, highlighting a potential tumor-driven tau pathology. Despite the relatively low pathological burden, our findings raise concern for systematic pTau seeding and spread with impact on cognition later on. The observed patterns of ADNC, in combination with microglial inflammation and DAI, underscore the need for further research to explore the clinical and potentially therapeutic implications of these pathological changes.

## Supplementary Material

vdaf057_suppl_Supplementary_Material
